# Recombinant IFN-γ1b Treatment in a Patient with Inherited IFN-γ Deficiency

**DOI:** 10.1007/s10875-024-01661-5

**Published:** 2024-02-16

**Authors:** Jérémie Rosain, Ayca Kiykim, Alexandre Michev, Yasemin Kendir-Demirkol, Darawan Rinchai, Jessica N. Peel, Hailun Li, Suheyla Ocak, Pinar Gokmirza Ozdemir, Tom Le Voyer, Quentin Philippot, Taushif Khan, Anna-Lena Neehus, Mélanie Migaud, Camille Soudée, Stéphanie Boisson-Dupuis, Nico Marr, Alessandro Borghesi, Jean-Laurent Casanova, Jacinta Bustamante

**Affiliations:** 1 Laboratory of Human Genetics of Infectious Diseases, Necker Branch, Necker Hospital for Sick Children, INSERM U1163, Paris, France; 2 University of Paris Cité, Imagine Institute, Paris, France; 3 Study Center for Primary Immunodeficiencies, Necker Hospital for Sick Children, Assistance Publique Hôpitaux de Paris (AP-HP), Paris, France; 4 Pediatric Allergy and Immunology, Cerrahpasa School of Medicine, Istanbul University-Cerrahpasa, Istanbul, Turkey; 5 Pediatric Clinic, IRCCS Policlinico “San Matteo” Foundation, University of Pavia, Pavia, Italy; 6 St. Giles Laboratory of Human Genetics of Infectious Diseases, Rockefeller Branch, Rockefeller University, New York, NY USA; 7 Department of Pediatric Genetics, Umraniye Education and Research Hospital, Istanbul, Turkey; 8 Pediatric Hematology and Oncology, Cerrahpasa School of Medicine, Istanbul University-Cerrahpasa, Istanbul, Turkey; 9 Pediatric Allergy and Immunology, School of Medicine, Trakya University, Edirne, Turkey; 10 Clinical Immunology Department, Saint-Louis Hospital, AP-HP, Paris, France; 11 Department of Immunology, Sidra Medicine, Doha, Qatar; 12 College of Health and Life Sciences, Hamad Bin Khalifa University, Doha, Qatar; 13 Neonatal Intensive Care Unit, Fondazione IRCCS Policlinico San Matteo, Pavia, Italy; 14 Howard Hughes Medical Institute, New York, NY USA; 15 Department of Pediatrics, Necker Hospital for Sick Children, AP-HP Paris, France

**Keywords:** Inborn error of immunity, Interferon-gamma, Mycobacterium, BCG

## Abstract

**Purpose:**

Inborn errors of IFN-γ immunity underlie Mendelian susceptibility to mycobacterial disease (MSMD). Twenty-two genes with products involved in the production of, or response to, IFN-γ and variants of which underlie MSMD have been identified. However, pathogenic variants of *IFNG* encoding a defective IFN-γ have been described in only two siblings, who both underwent hematopoietic stem cell transplantation (HCST).

**Methods:**

We characterized a new patient with MSMD by genetic, immunological, and clinical means. Therapeutic decisions were taken on the basis of these findings.

**Results:**

The patient was born to consanguineous Turkish parents and developed bacillus Calmette-Guérin (BCG) disease following vaccination at birth. Whole-exome sequencing revealed a homozygous private *IFNG* variant (c.224 T > C, p.F75S). Upon overexpression in recipient cells or constitutive expression in the patient’s cells, the mutant IFN-γ was produced within the cells but was not correctly folded or secreted. The patient was treated for 6 months with two or three antimycobacterial drugs only and then for 30 months with subcutaneous recombinant IFN-γ1b plus two antimycobacterial drugs. Treatment with IFN-γ1b finally normalized all biological parameters. The patient presented no recurrence of mycobacterial disease or other related infectious diseases. The treatment was well tolerated, without the production of detectable autoantibodies against IFN-γ.

**Conclusion:**

We describe a patient with a new form of autosomal recessive IFN-γ deficiency, with intracellular, but not extracellular IFN-γ. IFN-γ1b treatment appears to have been beneficial in this patient, with no recurrence of mycobacterial infection over a period of more than 30 months. This targeted treatment provides an alternative to HCST in patients with complete IFN-γ deficiency or at least an option to better control mycobacterial infection prior to HCST.

**Supplementary Information:**

The online version contains supplementary material available at 10.1007/s10875-024-01661-5.

## Introduction

Mendelian susceptibility to mycobacterial disease (MSMD) is defined as a selective susceptibility to weakly virulent mycobacteria [1–5]. Patients with this condition may also display infections with other intramacrophagic pathogens. MSMD can be “isolated” or “syndromic” if typically associated with other key infectious or non-infectious clinical phenotypes [1–5]. Since the recognition of MSMD 25 years ago, variants of 22 genes have been implicated in this condition (*CCR2*, *CYBB*, *IFNG*, *IFNGR1*, *IFNGR2*, *IL12B*, *IL12RB1*, *IL12RB2*, *IL23R*, *IRF1*, *IRF8*, *ISG15*, *JAK1*, *MCTS1*, *NEMO*, *RORC*, *SPPL2A*, *STAT1*, *TBX21*, *TYK2*, *USP18*, and *ZNFX1*) [1–9]. Allelic forms at these 22 loci define 44 genetic etiologies of MSMD. These genes encode proteins involved in the production of interferon-gamma (IFN-γ) (*IFNG*, *IL12B*, *IL12RB1*, *IL12RB2*, *IL23R*, *ISG15*, *MCTS1*, *RORC*, *TBX21*, *TYK2*), the response to IFN-γ (*CYBB*, *JAK1*, *IFNGR1*, *IFNGR2*, *IRF1*, *STAT1*, *USP18*), or both (*CCR2*, *IRF8*, *NEMO*, *SPPL2A*) [1–8]. ZNFX1 is the only exception, and the mechanistic connection between this gene and MSMD remains unclear [3].

The most severe forms of MSMD are caused by autosomal recessive (AR) complete deficiencies of IFN-γR1, IFN-γR2, IFN-γ, IRF1, STAT1, or IRF8 [2, 6, 10–30]. These six etiologies are truly Mendelian (i.e., with complete penetrance [31]) and are characterized by early-onset and recurrent infections with weakly virulent mycobacteria [2, 6, 10–30]. AR STAT1 and IRF8 deficiencies also underlie susceptibility to viral disease [29, 30]. AR complete IFN-γR1, IFN-γR2, IFN-γ, STAT1, IRF8, and IRF1 deficiencies underlie a complete absence of the IFN-γ-mediated antimycobacterial response [2, 6, 10–28, 30]. AR complete IFN-γR1 and IFN-γR2 deficiencies have been reported in more than 100 kindreds [10–28]; the patients’ cells are unable to respond to IFN-γ and hematopoietic stem cell transplantation (HCST) is the only curative treatment available [10–28]. AR complete IRF1 deficiency has been reported in two unrelated patients and is probably another indication for HCST [6]. AR complete STAT1 and IRF8 deficiencies have been reported in 24 and four patients, respectively, and HCST has been shown to improve outcome in these patients [29, 32, 33].

AR complete IFN-γ deficiency has been reported in only two related Lebanese patients living in Kuwait [2]. Both developed disseminated BCG disease (BCG-osis) a few weeks after vaccination with BCG at the ages of 4 and 5 months. One was treated with antimycobacterial drugs followed by HCST. She died at the age of 3 years, 9 days after HCST [2]. The other patient was initially treated with antimycobacterial drugs [2], subsequently underwent HCST, and is currently doing well (Waleed Al-Herz, *personal communication*). Despite the commercial availability of recombinant IFN-γ (IFN-γ1b), its efficacy and safety were not assessed in these patients. Patients with MSMD due to other inborn errors impairing the production of IFN-γ, such as IL-12p40 and IL-12Rβ1 deficiencies, have been shown to benefit from IFN-γ1b therapy [28, 34–40]. In this context, we describe a patient from Turkey with a novel form of AR complete IFN-γ deficiency who was treated for 30 months with recombinant IFN-γ1b.

## Results

### A Patient with Severe MSMD

**Fig. 1:**
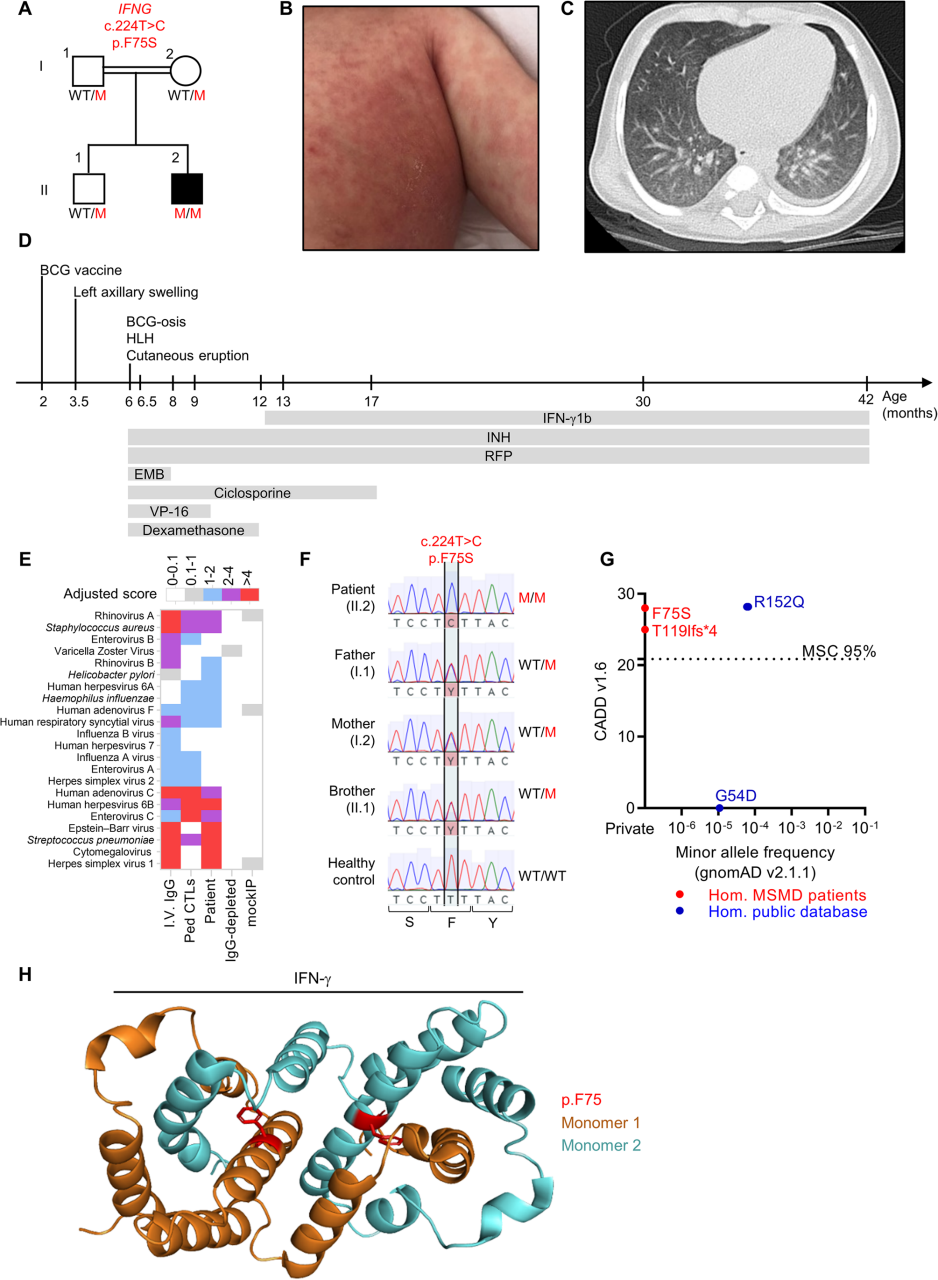
A new patient with a private rare biallelic variant of *IFNG*. **A** Pedigree of the kindred. Each generation is indicated by a Roman numeral, and each individual is indicated by an Arabic numeral. The patient is indicated by a black square. M, mutated; WT, wild-type.** B** Skin of the patient at the age of 6 months, with a maculopapular rash.** C** Computed tomography scan of the thorax of the patient at the age of 6 months showing pleural effusion in the left hemithorax. **D** Timeline of the follow-up of the patient with clinical events (top) and treatment (bottom). INH, isoniazid; RFP, rifampicin; EMB, ethambutol. **E** Antiviral antibody responses to species for which at least one sample tested seropositive by PhIP-Seq, based on stringent in-house cutoff values, color-coded as indicated. “cI.V.IgG” and “pediatric CTLs” correspond to the mean response for samples from pooled patients on IVIG (*n* = 8) and pediatric controls (*n* = 111) with a mean age of 9 years (SD = 2 years), respectively. A hierarchical clustering of samples based on antibodies directed against viruses is indicated at the top. **F** Electropherograms for the sequencing of representative *IFNG* nucleotide sequences from the patient, his relatives (heterozygous), and a healthy control (homozygous wild-type). **G** Combined annotation depletion-dependent (CADD) score *vs*. minor allele frequency (MAF) for variants in translated regions of *IFNG* found in the homozygous state either in public databases (blue) or in patients with AR complete IFN-γ deficiency. The 95% mutation significance cutoff (MSC) is indicated by a dotted line. **H** Crystal homodimer of IFN-γ (PDB = 6E3K [41]) showing the location of the p.F75 residue (red)

The patient (II.2), born in 2019, is the second child born to second-degree cousin parents of Turkish descent (Fig. 1A). He was born at full term after an uneventful pregnancy. His brother and both parents are healthy, but all three have a history of BCG lymphadenitis displaying spontaneous drainage without treatment. The patient was vaccinated with BCG (Russian strain) at the age of 2 months. The vaccine was injected into the left arm and, 6 weeks later, the patient presented axillary swelling on the left side. At the age of 6 months, he presented prolonged fever associated with left axillary lymphadenopathy (3 cm in size), hepatosplenomegaly, generalized maculopapular cutaneous rash (Fig. 1B), and failure to thrive. Computed tomography (CT) of the thorax revealed minimal pleural effusion in the left hemithorax (Fig. 1C). A biopsy of abdominal skin revealed mild orthohyperkeratosis in the epidermis, hypogranulosis, mild spongiosis, mild hyperplasia, and perivascular eosinophilic infiltration, but no pathogen was detected, even with periodic acid-Schiff (PAS) staining. No CD1a or langerin staining was detected. Blood cultures were negative for bacteria, mycobacteria, and fungi. Serological tests and PCR for cytomegalovirus (CMV) and Epstein-Barr virus (EBV) were negative. Laboratory tests revealed hypofibrinogenemia (1.2 g/L), hypertriglyceridemia (2.9 g/L), hyperferritinemia (> 2000 µg/L), anemia (5 g/dL), thrombocytopenia (57 G/L), and leukocytosis (32.9 G/L). The patient received multiple red blood cell transfusions for severe anemia. Peripheral blood lymphocytes and subsets were normal. Treatment with etoposide, dexamethasone, and cyclosporine was initiated due to a suspicion of hemophagocytic lymphohistiocytosis (HLH) (Fig. 1D). Etoposide, steroids, and cyclosporine were discontinued at the ages of 9, 12, and 17 months, respectively, due to improvement of the clinical and biological parameters of hemophagocytosis. Antimycobacterial treatment for suspected BCG-osis and targeted therapy of the patient with IFN-γ1b following genetic diagnosis are described below. The patient presented no life-threatening viral infections despite exposure to multiple DNA and RNA viruses, as demonstrated by VirScan on a blood sample collected at the age of 14 months (Fig. 1E). The patient had a severe form of MSMD, with BCG-osis, which was paradoxically associated with manifestations suggestive of HLH.

### The Patient Has a Private Biallelic Missense Variant of *IFNG*

Clinical whole-exome sequencing (WES) was performed on this patient and revealed a homozygous, single-nucleotide variant (c.224C > T) of *IFNG* (NM_000619.3). This variant was confirmed by Sanger sequencing, which also showed that the patient’s parents and brother were heterozygous (Fig. 1F). The c.224 T > C variant of *IFNG* is predicted to be missense (p.F75S). It is private (Fig. 1G) and absent from public databases of germline variants, including gnomAD v4.0.0 [42], BRAVO/TOPmed freeze 8 [43], the UK Biobank [44], ATAV [45], the Great Middle East database [46], the Turkish variome [47], and our in-house database of 25,000 individuals with various infectious diseases. This variant was predicted to be deleterious, with a combined annotation-dependent depletion (CADD) score even higher than the only other IFN-γ variant reported to date (p.T119Ifs*4) and above the mutation significance cutoff (MSC) [48, 49] (Fig. 1G). The p.F75 residue is located in α-helix C of IFN-γ (Fig. 1H), within a hydrophobic region highly conserved across species [50] (Supplementary Fig. 1A). The p.F75 residue is also conserved in cytokines from the IL-10 family [51], which have a similar structure to IFN-γ (Supplementary Fig. 1B). WES analysis of the patient identified no other candidate variants of known MSMD-causing genes or related genes. The patient was homozygous for a missense variant of *IFNG* predicted to be deleterious.

### On Overexpression, the IFN-γ p.F75S Variant Is Loss-of-Function

**Fig. 2:**
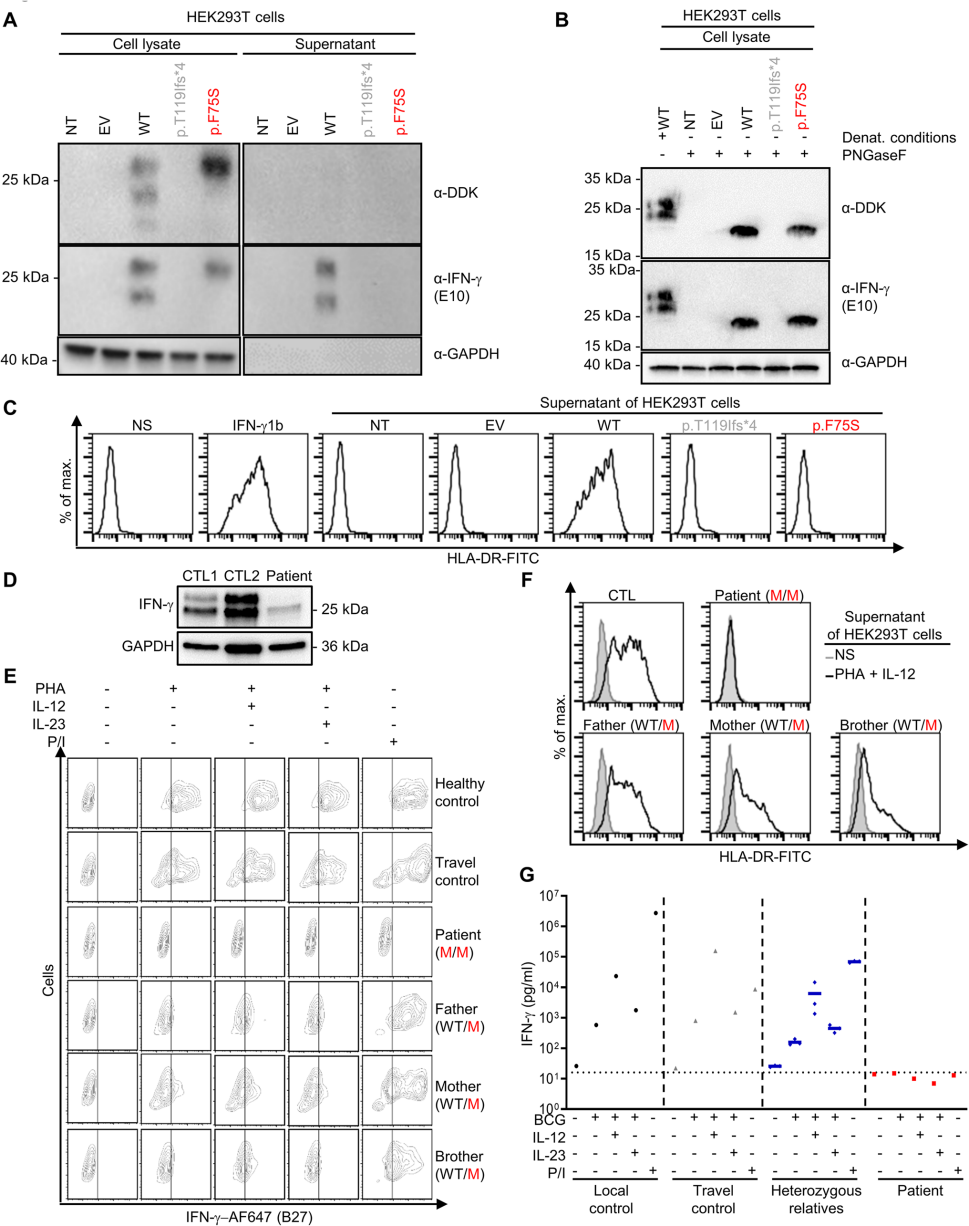
Autosomal recessive complete IFN-γ deficiency in the patient. **A** Western blot on total cell extracts from HEK293T cells left untransfected (NT, non-transfected) or transfected with the indicated plasmid, with an anti-DDK, anti-IFN-γ, or anti-GAPDH antibody used for detection. **B** Western blot on total cell extracts with and without prior treatment with PNGaseF from HEK293T cells untransfected or transfected with the plasmid indicated, with an anti-DDK, anti-IFN-γ, or anti-GAPDH antibody used for detection. **C** HLA-DR induction in SV40-fibroblasts with and without (NS, non-stimulated) treatment for 48 h with recombinant IFN-γ (IFN-γ1b) or with supernatant from untransfected HEK293T cells of HEK293T cells transfected with the plasmids indicated. **D** Western blot for IFN-γ and GAPDH on PHA-activated and anti-CD2/3/28 bead-activated blasts from two controls (CTLs) and the patient. **E** Intracellular flow cytometry for IFN-γ on PHA-activated T cells from the indicated patients or individuals after pretreatment with brefeldin in the indicated conditions (P/I, PMA/ionomycin). **F** HLA-DR induction in SV40-fibroblasts with and without (NS) treatment with supernatant from PHA-activated T-cell blasts from the indicated individuals or patients. **G** Secretion of IFN-γ, assessed in whole-blood assays, for the patient, the patient’s relatives, and healthy controls (local and travel), after activation with BCG (alone or in combination with IL-12, IL-23, or IFN-γ) or PMA/ionomycin (P/I)

We then used an overexpression system to assess the impact of the patient’s variant on IFN-γ protein production and function. We transiently transfected cultured human embryonic kidney 293 T (HEK293T) cells with an empty vector (EV), or a plasmid carrying the WT *IFNG* cDNA, or a cDNA corresponding to *IFNG* p.F75S or p.T119Ifs*4. This last variant was previously reported in two kindreds with AR complete IFN-γ deficiency [2] and served as a negative control. All plasmids were engineered with a C-terminal DDK tag. Western blotting detected WT IFN-γ protein products with apparent molecular weights (MW) of 15, 20, and 25 kDa in the cell lysate and supernatant, corresponding to the unglycosylated form and molecules with glycosylation at one or both sites, respectively [52] (Fig. 2A). In the cell lysate, the p.F75S IFN-γ protein detected had the same MW as the WT form with glycosylation at two sites. The same result was obtained with an antibody against the C-terminal DDK tag (Fig. 2A). The p.F75S IFN-γ protein was not detected in the supernatant of the transfected cells, whereas the WT IFN-γ was. As previously described [2], the protein encoded by the p.T119Ifs*4 variant was not detected in the cell lysate or supernatant. Treatment of the transfected cell lysate with PNGase-F, an endoglycosidase that removes N-linked glycans from glycoproteins, results in a similar MW for both the WT and p.F75S IFN-γ proteins, corresponding to the non-glycosylated form of the IFN-γ protein (Fig. 2B). The function of the mutant proteins was assessed by evaluating HLA-DR induction by supernatants from transfected HEK293T cells in SV-40-transformed fibroblasts (SV-40 fibroblasts). Supernatants from HEK293T cells transfected with the WT-*IFNG* cDNA induced HLA-DR expression on the surface of SV-40 fibroblasts from a healthy control, as in cells treated with IFN-γ1b, whereas supernatants from HEK293T cells transfected with the p.F75S and p.T119Ifs*4 variants did not (Fig. 2C). These results suggest that the biallelic variant found in the patient is loss-of-function due to impaired secretion and that MSMD in the patient is due to AR complete IFN-γ deficiency.

### The T Cells of the Patient Do Not Secrete Detectable Amounts of IFN-γ

We studied the production of IFN-γ in phytohemagglutinin (PHA)-activated blasts from controls and the patient. On a western blot of control denatured whole-cell lysate protein extracts, we detected IFN-γ proteins at the expected MW (Fig. 2D). In the same conditions, intracellular IFN-γ protein was also detected in PHA-activated blasts from the patient (Fig. 2D). We then studied the production of IFN-γ by flow cytometry with the B27 antibody targeting the tertiary structure of IFN-γ dimers [53]. PHA-activated blasts from healthy controls produced IFN-γ after additional stimulation with IL-12, IL-23, or phorbol myristate acetate/ionomycin (PMA/ionomycin) (Fig. 2E). By contrast, cells from the patient failed to produce detectable amounts of IFN-γ under the same conditions. Cells obtained from the heterozygous relatives of the patient produced smaller amounts of IFN-γ than the cells of healthy controls. The supernatant of PHA- and IL-12-activated cells from a healthy control and from heterozygous relatives induced HLA-DR expression in SV-40 fibroblasts, whereas the corresponding supernatant from the patient did not (Fig. 2F). We also measured cytokine secretion in whole-blood assays. After stimulation with BCG (alone or in combination with IL-12 or IL-23) or PMA/ionomycin, secreted IFN-γ was detected in whole blood from healthy controls (Fig. 2G). By contrast, the patient’s cells secreted no detectable IFN-γ under the same conditions (Fig. 2G) and smaller amounts of TNF (Supplementary Fig. 2A). The amounts of IL-12p40 produced in whole-blood assays were in the normal range after stimulation with BCG or BCG plus IFN-γ for the patient, his heterozygous relatives, and the controls (Supplementary Fig. 2B). Overall, these results suggest that the lymphoid cells of the patient produce intracellular IFN-γ that can be detected by western blotting but not by flow cytometry, i.e*.*, that the cells of the patient produce IFN-γ with an abnormal conformation.

### Efficacy, Pharmacology, and Safety of 30 Months of Treatment with IFN-γ1b

**Fig. 3:**
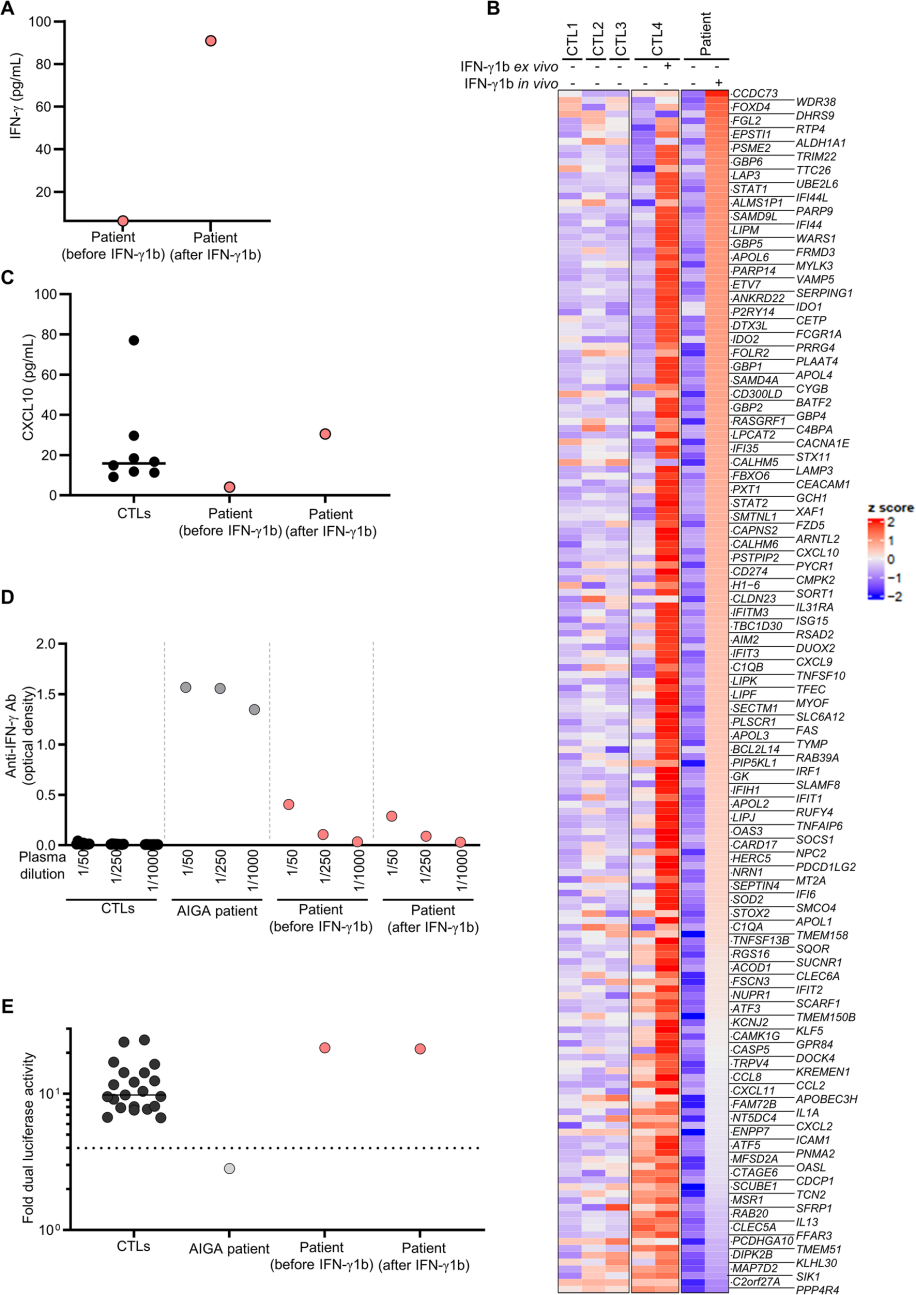
Efficacy and lack of immunogenicity of treatment with recombinant IFN-γ.** A** Quantification of IFN**-**γ levels in plasma from the patient before and 10 h after IFN-γ1b administration.** B** Sequencing of RNA extracted from the whole blood of three healthy controls (CTLs), healthy individuals with or without ex vivo treatment with IFN-γ1b for 8 h, and the patient before and 10 h after the subcutaneous administration of IFN-γ. **C** Quantification of IFN**-**γ levels in plasma from the patient before and 10 h after IFN-γ1b administration. Screening for autoantibodies against IFN-γ by **D** ELISA and **E** neutralization luciferase assays on plasma from the indicated controls or patients

The patient was initially treated for BCG-osis, between the ages of 6 and 8 months, with three antimycobacterial drugs (ethambutol, isoniazid, and rifampicin) (Fig. 1D). Ethambutol was discontinued after 2 months. The patient was treated with isoniazid and rifampicin only thereafter, between the ages of 8 and 12 months. The patient’s general state improved, leading to discharge from the hospital. The patients also displayed a decrease in ferritin levels and a normalization of fibrinogen levels. At the age of 12 months, subcutaneous recombinant human-modified IFN-γ (IFN-γ1b, Imukin), at a dose of 50 µg/m^2^ three times per week, was added to the treatment regimen based on rifampicin and isoniazid (Fig. 1D). On this treatment, the patient’s triglyceride and ferritin levels, which had remained high, finally normalized (Supplementary Table 2). The patient’s general condition remained good. The pharmacodynamic effects of IFN-γ1b do not appear to have been studied or reported by the industry during the development of the drug or in any of the subsequent clinical studies. We therefore performed RNA sequencing on fresh whole-blood samples collected before and 10 h after the subcutaneous administration of IFN-γ1b to the patient. IFN-γ levels were below the threshold of detection before IFN-γ1b administration and had reached 90 pg/mL 10-h post-administration (Fig. 3A). We also included whole blood from a healthy donor with and without ex vivo treatment with a high dose of IFN-γ1b as a positive control. In the positive controls and in the patient, we observed a positive induction of GAS-dependent myeloid interferon-stimulated genes [6], such as *IRF1*, *GBP4*, and *APOL3* (Fig. 3B). CXCL10 protein was also positively induced in the plasma of the patient (Fig. 3C). These results suggest that IFN-γ1b administration leads to the activation of myeloid cells activation in vivo. The administration of recombinant protein can lead to the generation of neutralizing autoantibodies (auto-Abs), as observed, for instance, in patients treated with recombinant type I IFNs [54–57]. We therefore also tested the patient for the production of auto-Abs against IFN-γ after 16 months of continuous treatment, with an immunoassay (ELISA) and a neutralization assay (luciferase assay). We found that no such antibodies were produced by the patient during treatment (Fig. 3D–E**)**). Consistent with the observed biological effect and the absence of auto-Abs, the patient was doing well on IFN-γ1b treatment and no recurrence of mycobacterial disease or any other infectious disease was observed. The treatment was well tolerated, as shown by follow-up data for temperature, biochemical tests, and whole-blood cell counts (Supplementary Table 2). The patient is currently 42 months old and is doing well on treatment with IFN-γ1b, isoniazid, and rifampicin.

## Discussion

We report an MSMD patient with a new form of AR complete IFN-γ deficiency, with conserved production of an abnormally folded intracellular IFN-γ protein that is not secreted. We also report the use of IFN-γ1b to treat this patient. IFN-γ1b treatment has previously been reported in several MSMD patients with normal or residual responses to IFN-γ, but not in the two previously reported patients with AR complete IFN-γ deficiency [28, 34–40]. To the best of our knowledge, this is also the first report of a patient with an inherited deficiency of a cytokine treated with the corresponding recombinant cytokine as replacement therapy. This treatment appears to have been successful, as a clinical and biological improvement in the patient’s condition was observed when IFN-γ was added to the therapeutic regimen. No recurrence of mycobacterial disease was observed. Nevertheless, the patient was also treated, initially with three and subsequently with two antimycobacterial drugs over a period of 6 months, which may have contributed to his clinical remission. However, an analysis of the natural course of disease of all previously reported patients with a complete lack of IFN-γ activity due to AR complete IFN-γ, IFN-γR1, IFN-γR2, IRF1, or STAT1 deficiency revealed that these patients were unable to control mycobacterial infections and that treatment regimens including fewer than four drugs were unable to prevent recurrences or new mycobacterial infections in these patients [2, 6, 10–29]. Even with four antibiotics, these patients rarely achieve clinical remission for more than a few months [39]. Together, these findings suggest that IFN-γ1b was effective in the patient reported here.

The patient reported here also displayed good tolerance to the treatment, with no clinical or biological adverse effects. In particular, IFN-γ administration did not lead to an inflammatory syndrome with a high temperature or to a recurrence of HLH-like manifestations. The main risk of providing exogenous protein to compensate for the genetic deficiency of the endogenous protein is the development of neutralizing antibodies, which have been observed in a subset of patients with inherited deficiencies of coagulation proteins (e.g., factors VII, VIII, and IX) [58], ß-glucocerebrosidase factor deficiency underlying Gaucher disease [59], and adenosine deaminase deficiency underlying severe combined immunodeficiency [60]. No neutralizing anti-IFN-γ auto-Abs were detected in the serum of the patient during the 30 months of treatment administration. We suggest monitoring the efficacy and safety of IFN-γ1b therapy by regular clinical, radiological, and biological work-up. A longer follow-up period is required, but IFN-γ1b treatment appears, so far, to have been a safe and effective treatment for complete IFN-γ deficiency in this patient. This treatment may be a useful alternative to HCST in patients with complete IFN-γ deficiency or at least an option for curing the patient of mycobacterial disease before performing HCST.

## Materials and Methods

### Patient

The patient was born and is living in Turkey. Written consent was obtained in Turkey, in accordance with local regulations and with institutional review board (IRB) approval. Experiments were conducted in France, Qatar, and the USA, in accordance with local regulations and with the approval of the IRBs of the Rockefeller University and INSERM, for the USA and France, respectively. Healthy controls were recruited in France and Turkey.

### Clinical WES

Clinical WES, covering 5000 genes, was performed for the patient. Genomic DNA was extracted from EDTA-treated peripheral blood with a semi-automated robot, in accordance with the manufacturer’s recommendations (Qiagen). Spectrophotometry (Nanodrop 2000, Thermo Fisher Scientific, USA) and fluorometrics (Qubit v3.0, Thermo Fisher Scientific, USA) were used to obtain absorbance ratios at 260/280 nm and 260/230 nm for the analysis of DNA concentration and quality, respectively. The capture-based Clinical Exome Solution Kit from Sophia Genetics was used during the preparation of samples for next-generation sequencing (NGS). NextSeq 500 (Illumina, USA) was used. Data quality control, alignment, variant calling, and variant annotations were performed with the Sophia DDM analysis tool (version 5.2). NCBI Build37 (hg19) was used as a reference library for human genome sequences. Variants located within 10 base pairs on either side of the targeted exons with a minimum read depth of 50 × were selected. Variants outside these regions, those in homopolymer regions, and exonic variants with a variant fraction below 20% were considered false positives and were not analyzed. All variants were manually inspected with the Integrative Genomics Viewer (IGV) visualization tool.

### Cells

HEK293T cells and SV40-fibroblasts were cultured in Dulbecco/Vogt modified Eagle’s minimal essential medium (DMEM, #61,965,059, Gibco) supplemented with decomplemented 10% fetal bovine serum (FBS, #10,270,098, Gibco). PHA-activated T cells were cultured in Roswell Park Memorial Institute medium (RPMI 1640, # 61,870,044, Gibco) supplemented with 10% decomplemented fetal bovine serum (FBS). PHA-activated T-cell blasts were cultured in ImmunoCult-XF T Cell Exp Medium (#10,981, Stemcell) in the presence of IL-2 and were primed every 2 weeks with ImmunoCult Human CD3/CD28/CD2 T-Cell Activator (#10,970, Stemcell). All cells were grown at 37 °C, under an atmosphere containing 5% CO_2_.

### Western Blotting

Western blotting was performed as previously described [2]. Briefly, total protein extracts were prepared by mixing cells with modified radioimmunoprecipitation assay buffer supplemented with protease inhibitors (EDTA-free Complete, Roche) and phosphatase inhibitor cocktail (PhosphoStop, Roche), 0.1 mM dithiothreitol (DTT; Life Technologies), 10^–3^ mM Na_3_VO_4_, and 1 mM PMSF and incubating the resulting mixture for 40 min on ice. Protein extracts were subjected to electrophoresis in 12% acrylamide precast gels (Biorad) and the resulting bands were transferred onto nitrocellulose membranes (Biorad) by semi-dry transfer. Membranes were then incubated with mouse monoclonal anti-human IFN-γ N-terminus (#sc-373727, clone E-10, Santa Cruz Biotechnology), anti-GAPDH (#2118, clone 14C10, Cell Signaling Technology), or HRP-conjugated anti-DDK (clone M2, Sigma-Aldrich) antibodies. The anti-IFN-γ antibody was detected with HRP-conjugated goat anti-mouse secondary antibodies (Bio-Rad).

### Flow Cytometry

For HLA-DR induction, SV40-fibroblasts were plated at a density of 200,000 cells per well, in six-well plates, with 2 mL DMEM-10% FBS per well, and were left unstimulated or were stimulated the following day with 10^3^ IU/mL IFN-γ1b (IFN-γ, Imukin, Horizon Pharma or Medipha Santé), or 100 µL of supernatant from untransfected HEK293T cells of HEK293T cells transfected with indicated plasmids, or 100 µL of supernatant of PHA-activated T-cell blasts. The cells were harvested by trypsin digestion 48 h after stimulation and stained as described below.

### Whole-Blood Activation ELISA for Cytokines

Venous blood samples from healthy controls and the patient were collected in heparin-containing collection tubes [61, 62]. These samples were diluted 1:2 in RPMI 1640 (Gibco) supplemented with 100 IU/mL penicillin and 100 μg/mL streptomycin (Gibco). We then dispensed 1 mL of each diluted blood sample into each of five wells (1 mL/well) of a 48-well plate (Nunc). These samples were incubated for 48 h at 37 °C, under an atmosphere containing 5% CO_2_/95% air and under various activation conditions: with medium alone, with live BCG (*M. bovis*-BCG, Pasteur substrain) at a MOI of 20 BCG cells/leukocyte, or with BCG plus recombinant (rh) IL-12 (20 ng/ml; R&D Systems) or BCG plus IFN-γ (Imukin). The supernatants were then collected and subjected to ELISA.

### Phage Immunoprecipitation-Sequencing (PhIP-Seq)

A plasma sample was collected from the patient at the age of 14 months. The patient was never treated with exogenous IgG. For antibody profiling by phage immunoprecipitation-sequencing (PhIP-Seq) [63], plasma samples from the patient and controls were assayed and data were analyzed as previously described [2, 64], but with the following modifications. We calculated species-specific significance cutoff values to estimate the minimum number of enriched, non-homologous peptides required to consider a sample seropositive (as previously described [63]) with an in-house dataset and a generalized linear model. For each sample, we calculated virus-specific scores by dividing the counts of enriched, non-homologous peptides by the estimated score cutoff. These adjusted virus scores were used in the heatmap plot. In addition to studying the patient reported here, we also analyzed and plotted the mean antibody responses for a pediatric control cohort of lean individuals without infectious or immunological disease (*n* = 111; age range, 7 to 15 years; median age, 11.0 years) described in a previous study [65, 66]. Pooled human plasma used for IVIg (Privigen® CSL Behring AG) and human IgG-depleted serum (Molecular Innovations, Inc.) served as additional controls. All research on human subjects was performed after informed written consent had been obtained or after the samples had been rendered anonymous. The procedures were approved by the Institutional Research Ethics Boards of Sidra Medicine.

### Cytokine Determination by ELISA and Flow Cytometry

Supernatants from whole-blood stimulation experiments were assessed for the determination of IL-12p40 (#DP400, R&D Systems), IFN-γ (#430,116, BioLegend), or TNF (Sanquin), in accordance with the manufacturer’s protocol. IFN-γ levels in plasma were determined with LEGENDplex™ Human Inflammation Panel 1 (#740,809, BioLegend).

### RNA Sequencing

Whole-blood samples were collected into PAXgene tubes (#762,165, Qiagen). Blood from the patient was collected directly into PAXgene tubes before and after 10 h of subcutaneous IFN-γ1b (Imukin at a dose of 50 µg/m^2^) administration. Blood from three healthy adult controls was also collected directly into PAXgene tubes. As a positive control for IFN-γ stimulation, blood from a fourth control was collected into heparin-containing tubes, with or without a saturating dose of IFN-γ1b (10^5^ IU/mL), and the tubes were incubated for 8 h at 37 °C, after which 2.5 mL of blood was transferred to a PAXgene tube. Blood was stored at − 80 °C and RNA was extracted with the PAXgene Blood RNA Kit (#762,174, Qiagen). We performed mRNA sequencing on an Illumina Nextseq platform, with a read length of 100 bp and a read depth of 80 Mread. All FASTQ files passed quality control and were aligned with the GRCh38 reference genome with STAR (2.6.1d). BAM files were converted into a raw count expression matrix with feature count. Raw count data were normalized with DEseq2 [67]. The ensemble IDs targeting multiple genes were collapsed (average), and a final data matrix gene was generated for downstream analysis. For differential expression analysis, the genes displaying significant differential expression were selected according to the following criteria: FDR ≤ 0.05 and |log2(FoldChange)|≥ 1.5. A heatmap representing classical monocyte transcript abundance profiles (*z*-score-scaled log_2_ normalized counts) was generated with ComplexHeatmap [68].

### Screening for Auto-Abs Against IFN-γ by ELISA

Plasma from the patient and healthy controls was screened by ELISA for anti-cytokine auto-Abs, as previously described [33]. Maxisorp 96-well ELISA plates (Maxisorp; Nunc) were coated by incubation overnight at 4 °C with 1 µg/ml recombinant human IFN-γ (R&D Systems). Plates were then washed (PBS/Tween 0.005%), blocked by incubation with the same buffer supplemented with 5% nonfat milk powder, washed, and incubated with 1:1000, 1:250, and 1:50 dilutions of serum samples from the patient or controls for 2 h at room temperature. The plates were then thoroughly washed and horseradish peroxidase (HRP)–conjugated Fc-specific IgG fractions from polyclonal goat antiserum against human IgG (Nordic Immunological Laboratories) were added to a final concentration of 1 µg/mL. The plates were incubated for 1 h at room temperature and washed again. Substrate was added and the OD was measured (Victor X4™, Perkin Elmer).

### Screening for Auto-Abs Against IFN-γ in Luciferase Assays

The blocking activity of anti–IFN-γ auto-Abs was assessed by determining reporter luciferase activity. Briefly, *IFNAR1*^−/−^ HeLa cells were transfected with a plasmid containing the firefly luciferase gene under the control of six tandem repeats of the human *GAS* promoter and a plasmid constitutively expressing *Renilla* luciferase for normalization (#CCS-009L, Cignal reporter assay kits, Qiagen). Cells were transfected in the presence of the Lipofectamine LTX and Plus transfection reagents (Invitrogen, reference number 15338–100) for 24 h. Cells in Dulbecco’s modified Eagle medium (DMEM, Thermo Fisher Scientific) supplemented with 5% FBS and 10% healthy control or patient serum/plasma (after inactivation at 56 °C for 20 min) were stimulated with 200 pg/ml IFN-γ (Imukin, 2 × 10^6^ UI (0.1 mg)) for 16 h at 37 °C. Finally, cells were lysed for 10 min at room temperature, and luciferase levels were measured with the Dual-Glo Luciferase Assay System (#E2940, Promega) according to the manufacturer’s protocol. Luminescence intensity was measured with a SpectraMax Id3 (Molecular Devices). Firefly luciferase activity values were normalized against *Renilla* luciferase activity values. These values were then normalized against the fold-change induction for pooled healthy serum (#H4522-20ML, Sigma) in the presence of 200 pg IFN-γ relative to non-stimulated, pooled healthy serum. Samples were considered neutralizing if luciferase induction, normalized against *Renilla* luciferase activity, was below 10% of the mean value for controls tested the same day.

### Supplementary Information

Below is the link to the electronic supplementary material.

Supplementary file1 (DOCX 276 KB)

## Data Availability

All data are either included in the manuscript or available upon request.
