# Supplementary material for: Recombinant IFN-γ1b Treatment in a Patient with Inherited IFN-γ Deficiency
Source: J Clin Immunol. 2024 Feb 16;44(3):62. doi: 10.1007/s10875-024-01661-5 (PMC10873451; doi:10.1007/s10875-024-01661-5)
Supplement: Supplementary file 1 — Supplementary file1 (DOCX 276 KB) [file 10875_2024_1661_MOESM1_ESM.docx]

**Supplementary figure legends**

**Supplementary Figure 1 – (A)** CLUSTALW alignment of the p.F75 residue and flanking residues with homologous IFN-γ proteins. **(B)** CLUSTALW alignment of the p.F75 residue and flanking residues with paralogous IFN-γ proteins.

**Supplementary Figure 2 -** Secretion of TNF **(A)** and IL12p40 **(B)** assessed in assays on whole blood from the patient, the patient’s relatives and healthy controls (local and travel), after activation with BCG (alone or in combination with IL-12, IL-23 or IFN-γ1b ) or PMA/ionomycin (P/I).


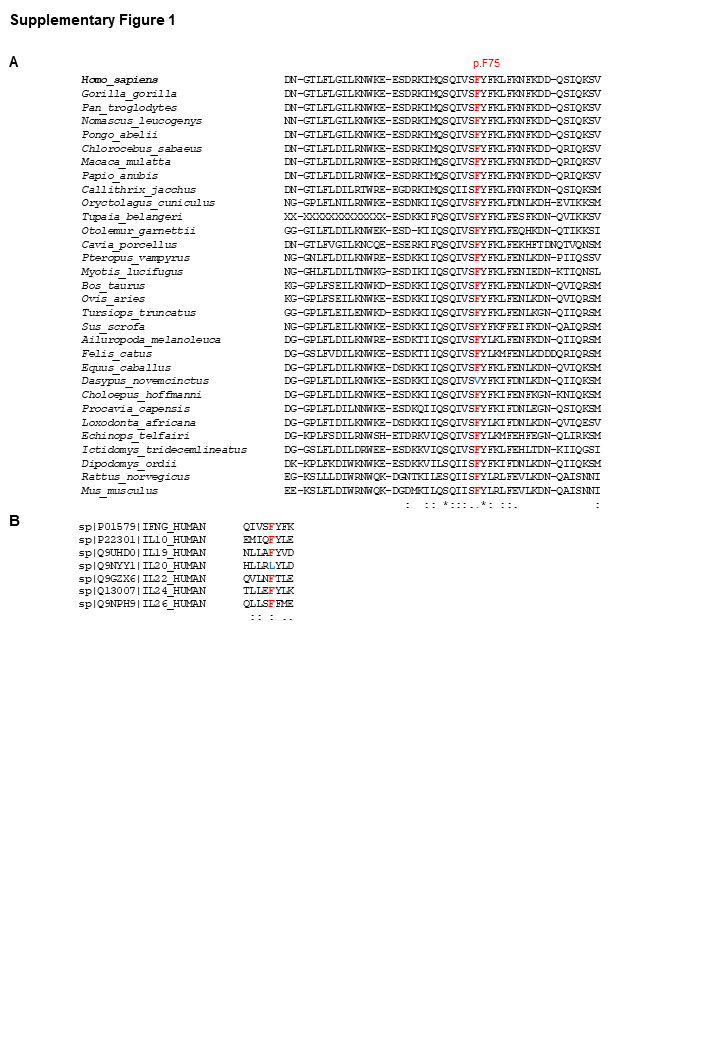

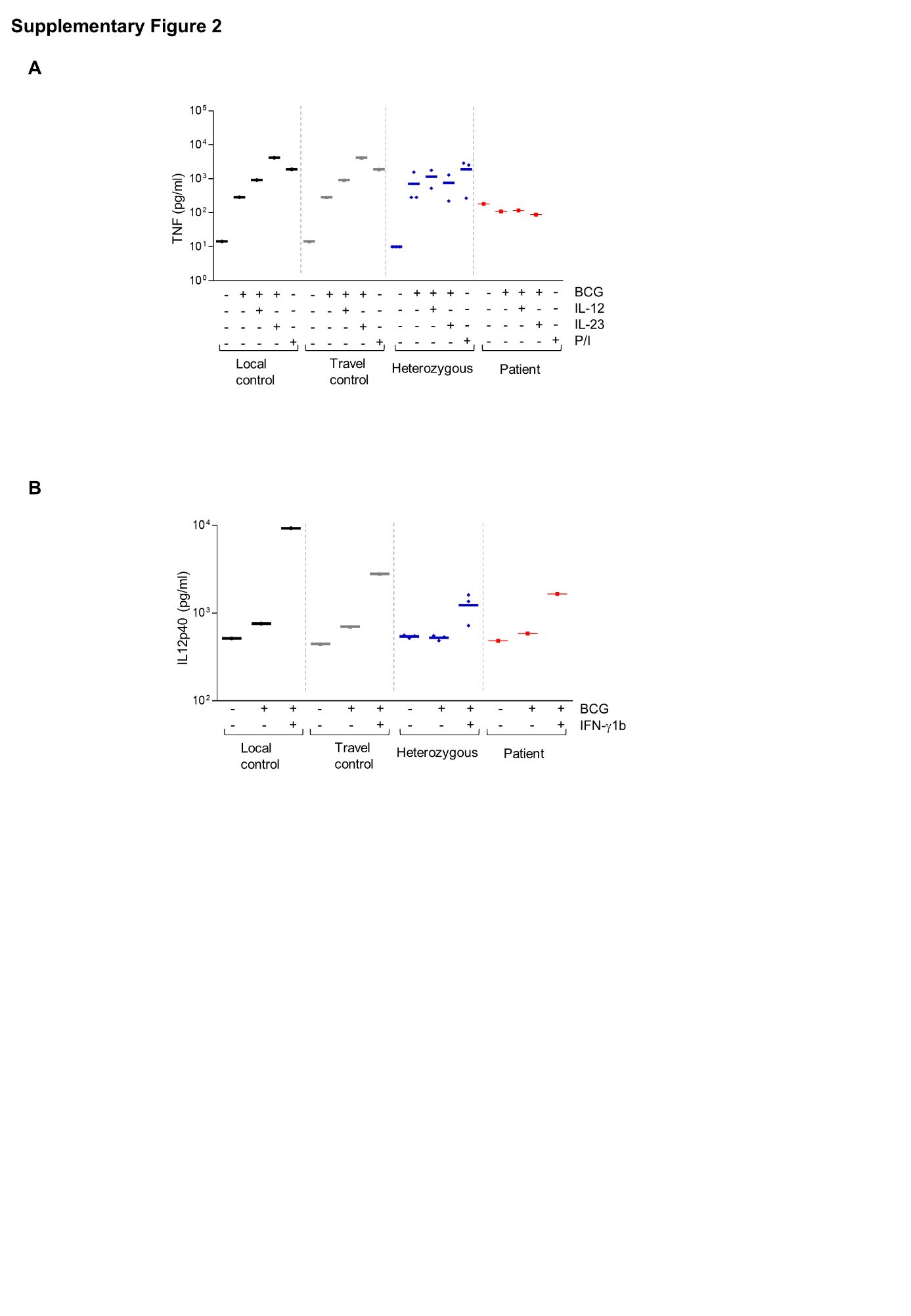


**Supplementary Tables**

**Supplementary Table 1 – Patient’s lymphocytes and subsets in peripheral blood: comparison with an age-matched healthy control**

| Cell type | Patient (11 months) | Age-matched control range |
| --- | --- | --- |
| Lymphocytes | 5.49 x 10^3^ /µl |  |
| T cells |  |  |
| CD3+ | 66 %  3.62 x 10^3^ /µl | 49-76 %  1.90-5.90 x 10^3^ /µl |
| CD4+ | 42 %  2.30 x 10^3^ /µl | 31-56 %  1.40-4.30 x 10^3^ /µl |
| CD8+ | 19 %  1.04 x 10^3^ /µl | 12-24 %  0.50-1.70 x 10^3^ /µl |
| B cells |  |  |
| CD19+ | 26 %  1.43 x 10^3^ /µl | 12.40-33.60 %  0.52-2.15 x 10^3^ /µl |
| NK cells |  |  |
| CD16+CD56+ | 5 %  0.27 x 10^3^ /µl | 3-15 %  0.16-0.95 x 10^3^ /µl |

**Supplementary Table 2 – Follow-up of the patient’s temperature and blood test findings before and after the administration of IFN-γ1b.** N=normal range. ND=non-determined.

| Age (months old) | 6 | 8 | 11 | 12 | 14 | 15 | 18 | 21 | 23 | 27 | 30 | 33 | 36 |
| --- | --- | --- | --- | --- | --- | --- | --- | --- | --- | --- | --- | --- | --- |
| Rifampicin | + | + | + | + | + | + | + | + | + | + | + | + | + |
| Isoniazid | + | + | + | + | + | + | + | + | + | + | + | + | + |
| Ethambutol | + | - | - | - | - | - | - | - | - | - | - | - | - |
| IFN-γ1b | - | - | - | + | + | + | + | + | + | + | + | + | + |
| Temperature (°C) N=36-37.5 | 38.5 | 36.5 | 36.2 | 36.4 | 36 | 37 | 36.5 | 36.8 | 37 | 36.1 | 36.3 | 36 | 37 |
| While blood cells count (G/L)  N=5.2-12.4 | **11.4** | **10** | 10.4 | 10.9 | 10 | 8.9 | 11.6 | 7.3 | 9.8 | 10.5 | 11.9 | 7.1 | 7.6 |
| Neutrophil count (G/L) N=1.9-8 | 5.5 | 4.2 | 2.9 | 2.5 | 2.2 | 2.4 | 2 | 1.7 | 2.3 | 3.1 | 4.9 | 1.6 | 2.8 |
| Eosinophil count (G/L)  N<1 G/L | **2.4** | 0.6 | 0.3 | 0.4 | 0.4 | 0.3 | **1.2** | 0.4 | 0.9 | 0.6 | 0.4 | 0.6 | 0.9 |
| Basophil count (G/L)  N<0.1 | **0.2** | 0.1 | 0.1 | 0.1 | 0.1 | 1.2 | 0.1 | 0 | 0.1 | 0.1 | 0.1 | 0 | 0.1 |
| Monocyte count (G/L)  N=0-1 | **0.8** | **1.2** | 0.6 | 0.6 | 0.7 | 0.7 | 0.9 | 0.6 | 0.7 | 1 | 0.6 | 0.4 | 0.7 |
| Lymphocytes count (G/L) N=0.9-5.2 | 4.6 | 4.6 | 6.5 | **7.3** | **6.7** | **5.5** | 7.5 | 4.6 | **5.8** | **6.1** | **5.8** | 4.5 | 3.5 |
| Hemoglobin (g/dL) N=11.2-18 | 8.8 | 12.2 | 14.3 | 13.9 | 15 | 15 | 14.7 | 14.2 | 15 | 14.3 | 13.3 | 14 | 14 |
| Platelets (G/L) N=130-400 | 43 | 443 | 357 | 394 | **436** | 367 | 430 | 369 | 335 | 369 | **517** | 279 | 371 |
| AST (IU/L) N=10-40 | 8 | 37 | 39 | 35 | 40 | **44** | 38 | 34 | 30 | 42 | 29 | 33 | 33 |
| ALT (IU/L N=10-40 | 12 | 41 | 29 | 28 | 35 | 29 | 29 | 19 | 20 | 18 | 22 | 18 | 13 |
| Urea (mg/dL) N=10-50 | 15 | 11 | 12 | 15 | 30 | 38 | 35 | 32 | NA | 27 | NA | 23 | 32 |
| Creatinine (mg/dL) N=0.3-1.1 | 0.07 | 0.11 | **0.19** | NA | **0.2** | **0.2** | **0.22** | **0.22** | NA | **0.26** | NA | **0.2** | 0.4 |
| Erythrocyte sedimentation rate (mm/h) N=0-15 | 6 | 9 | 2 | 4 | 4 | 3 | ND | ND | 3 | 4 | 5 | 5 | 4 |
| Ferritine (µg/L) N=30-400 | **1992** | **884** | **522** | 265 | 262 | 206 | 136 | 107 | 95 | 48.1 | NA | NA | 39 |
| Fibrinogen (g/L)  N=1.8-3.5 | **1.2** | 3.7 | 2.3 | 2.1 | 2.3 | 2.7 | 3.3 | 2.7 | 2.7 | 1.9 | 2.4 | 2.4 | 2.5 |
| Triglycerides (g/L)  N=0.3-1.35 | **3.0** | **2.6** | 1.5 | **3.1** | 1.3 | 0.8 | 1.3 | 1.5 | 0.9 | 1.1 | 2.6 | 1.4 | 1.3 |
